# Exposure and work‐related factors in subjects with hand eczema: Data from a cross‐sectional questionnaire within the Lifelines Cohort Study

**DOI:** 10.1111/cod.14066

**Published:** 2022-03-15

**Authors:** Marjolein J. Brands, Laura Loman, Marie L.A. Schuttelaar

**Affiliations:** ^1^ Department of Dermatology, University of Groningen University Medical Center Groningen Groningen The Netherlands

**Keywords:** general population, hand dermatitis, hand eczema, non‐occupational wet exposure, occupational wet exposure, occupation, socioeconomic factors, work‐related factors

## Abstract

**Background:**

Hand eczema (HE) is the most frequently occurring occupational skin disease. However, studies on non‐occupational wet exposure, occupations not considered as high‐risk, and socioeconomic factors regarding HE are scarce.

**Objectives:**

To investigate the association between HE and occupational and non‐occupational wet exposure and work‐related factors in the Dutch general population.

**Methods:**

Within the Lifelines Cohort Study, participants with HE were identified by a digital, add‐on questionnaire that included questions regarding exposure. Data on work‐related and socioeconomic factors were collected from baseline.

**Results:**

Overall, 57 046 participants (42.0%) were included. Occupational and non‐occupational wet exposure were positively associated with HE in the past year (odds ratios (ORs) 1.35, [95% confidence interval (CI): 1.22–1.49] and 1.34, [95%CI: 1.17‐1.53], respectively). Positive associations for high‐risk occupations (OR 1.20, [95%CI: 1.06‐1.36] for personal care workers in health services and OR 1.25, [95%CI: 1.06‐1.48] for nursing and midwifery professionals), occupations not considered as high‐risk (OR 1.19, [95%CI: 1.03‐1.39] for legal, social and religious associate professionals) and higher levels of education were found (OR 1.17, [95%CI: 1.04‐1.32] and OR 1.18, [95%CI: 1.04‐1.34] for middle and high level, respectively).

**Conclusion:**

Preventive strategies for HE should focus on avoidance of all wet exposure, regardless of origin. In addition, job tasks instead of job title should be taken into account. As previous results on the association between HE and socioeconomic factors differ, future research should focus on a uniform definition of socioeconomic status.

## Highlights

- Preventive strategies for hand eczema should focus on avoidance of all exposure to wet activities, regardless of origin.
- Job tasks instead of job title should be considered when counseling patients with hand eczema.
- Future research on the association between hand eczema and socioeconomic factors should focus on a uniform definition of socioeconomic status.

## 1 INTRODUCTION

Hand eczema (HE) is a common skin disease, with a 1‐year prevalence of up to 10%, and a lifetime prevalence of nearly 15% in the general population.^1^ The course of HE is often chronic and relapsing, with negative consequences on quality of life, working ability, career prospects, and participation in social activities of those affected.^2^, ^3^ In addition, it is considered one of the most frequent occupational skin diseases.^4^ The lifetime prevalence of HE related to work ranges from 1%– 5% in the general population.^5^, ^6^

An important risk factor associated with HE is wet work.^7^ The association between HE and wet work has been studied extensively.^7^, ^8^, ^9^, ^10^ Most previous studies have focused on occupational exposure, whereas studies on non‐occupational exposure are scarce. However, the hands are exposed to water and other irritants not only at work, but also during leisure time.^11^ Therefore, non‐occupational exposure is considered to also contribute to the risk of HE.^7^, ^8^, ^9^, ^11^

Several occupations, such as cleaning and medical or nursing work, are regarded as occupations with a high probability of causing HE, because of the exposure to wet work.^7^, ^12^ The association between high‐risk occupations and HE has been studied intensively, however, less is known about the association between HE and occupations not considered as high‐risk occupations.^5^, ^13^, ^14^ Occupational risk factors could contribute to a higher risk of HE, even when not working in a high‐risk occupation. It is possible that, based on the job title, exposure would not be directly expected, but certain tasks involved in the job could include exposure.

Besides occupational‐related factors, the possible association between HE and socioeconomic status (SES) has been reported.^15^, ^16^, ^17^, ^18^, ^19^ For instance, educational attainment, income, or a combination of these socioeconomic factors have been studied in the association with HE.^15^, ^16^, ^17^, ^19^ Since data on SES are limited, further research on the association between HE and different socioeconomic measures is needed.

The aim of the present study was to investigate the association between HE and occupational and non‐occupational wet exposure, and work‐related and socioeconomic factors in the general population of the Northern Netherlands.

## 2 METHODS

### 2.1 Design, setting and participants

The study was a cross‐sectional, add‐on study within the (LCS).^20^ The Lifelines Cohort Study is a multi‐disciplinary, prospective, population‐based cohort study, examining the health and health‐related behaviours of 167 729 persons living in the Northern Netherlands, in a unique three‐generation design. It employs a broad range of investigative procedures in assessing the biomedical, sociodemographic, behavioural, physical, and psychological factors that contribute to the health and disease of the general population, with a special focus on multi‐morbidity and complex genetics. The Lifelines adult study population is broadly representative of the adult population of the Northern Netherlands.^21^ Data collection in the LCS was conducted according to the guidelines of the Declaration of Helsinki, and all procedures were approved by the Medical Ethics Committee of the University Medical Center Groningen (reference number: METc 2007/152, reference number current add‐on study: METc 2019/571). A self‐administrable digital, add‐on questionnaire regarding dermatological diseases was developed and sent to 135 950 Lifelines participants aged 18 years and older. Of them, 58 198 participants responded (42.8%). To the question regarding the lifetime prevalence of HE, 57 046 participants (42.0%), aged 18 years and older at baseline, responded and were included in the current analysis. Details on prevalence and severity of HE in the whole study population were described previously.^22^

### 2.2 Questionnaire

A digital questionnaire was sent out between February 6 and March 2, 2020 and a digital reminder to non‐responders was sent within the following 10 weeks. The questionnaire was available until May 15, 2020. The lifetime and 1‐year prevalence of HE were identified by the questions “Have you ever (now or in the past) had hand eczema?” and “Have you had hand eczema in the past 12 months?”, respectively (based on the Nordic Occupational Skin Questionnaire (NOSQ‐2002; QD1 adjusted)).^23^ Physician diagnosed atopic dermatitis (AD) was identified by the question “Have you ever been diagnosed with atopic dermatitis or atopic eczema by a doctor?”.^24^ Data regarding HE, physician diagnosed AD, contact allergy, wet exposure, and high‐risk occupations were extracted from the add‐on questionnaire. Data on sex, age, occupation, work‐related, and socioeconomic factors were collected from baseline assessments that took place between 2006 and 2013. The occupational factors assessed in the current study included employment status and hours of work per week. The socioeconomic factors included were educational attainment, SES, and income. Details about the questions used in the current study can be found in Appendix S1.

### 2.3 Occupational and non‐occupational wet exposure

Occupational wet exposure was determined according to the German “Technische Regeln für Gefahrstoffe” (TRGS) 401 criteria.^7^, ^12^, ^25^ It was based on three questions regarding exposure of the hands on an average working day at the workplace (occupational wet exposure), concerning: (i) hours of direct contact with water, fluids, and/or moist products; (ii) hours of wearing gloves that are impermeable to fluids; and (iii) the frequency of hand washing. The same questions were asked regarding an average day at home (non‐occupational wet exposure). Wet exposure was defined as: (i) activities where individuals have to immerse their hands in liquids for >2 hours daily, or wear (occlusive) gloves for >2 hours daily, or wash their hands >20 times a day, or (ii) a combination of two of the following: activities where individuals have to immerse their hands in liquids for 1‐2 hours daily, wear (occlusive) gloves for 1‐2 hours daily, or wash their hands 10‐20 times a day. In addition, a variable concerning overall wet exposure was defined, by combining all six questions regarding both occupational and non‐occupational wet exposure. Details about the criteria used to define the variables concerning wet work exposure can also be found in Appendix S1.

### 2.4 Occupational groups

Participants’ professions at baseline were coded according to the International Standard Classification of Occupations, version 2008 (ISCO‐08) and aggregated into major and minor groups, based on the three‐digit level.^26^ ISCO‐08 major groups with at least 75 cases of HE were included. In addition, a list of high‐risk‐occupations, defined as having evident exposure to allergens, or wet work and/or friction, with a high probability of developing HE, was included in the add‐on questionnaire to determine 1‐year prevalence of HE while working in one of these high‐risk occupations.^12^, ^27^, ^28^, ^29^, ^30^, ^31^, ^32^

### 2.5 Educational attainment

Educational level was classified according to the International Standard Classification of Education^33^ and classified as low (no education, primary education, lower or preparatory secondary vocational education, junior general secondary education); medium (secondary vocational education or work‐based learning pathway, senior general secondary education, pre‐university secondary education); and high educational attainment (higher vocational education or university education).

### 2.6 Socioeconomic status

Socioeconomic status was based on neighbourhood SES scores according to Statistics Netherlands and the Netherlands Institute for Social Research, which were linked to the postal codes of the participants of the Lifelines cohort. The status scores for each postal code within the Netherlands are based on the inhabitants' average income, educational level, and job perspective. Scores given to postal codes range from −8 to +3, with a score of ~ 0 representing an average SES in the Netherlands.^34^ In the current study, SES scores were grouped into three main categories, defined as low SES (score below ‐1), middle SES (score between −1 and + 1), and high SES (score above +1).

### 2.7 Income

Net household income was classified into eight categories: less than 750; 750–1000; 1000–1500; 1500–2000; 2000–2500; 2500–3000; 3000–3500, and ˃ 3500 euros per month.

### 2.8 Employment status

Employment status was divided in four categories: unemployed, retired (including early retirement), unfit for work, and employed. Individual participants were placed in only one category.

### 2.9 Working hours

The number of working hours per week was reported as a discrete variable and was divided into six categories: 1‐8; 9‐16; 17‐24; 25‐32; 33‐40, and > 40 work hours per week. Values exceeding ≥ 80 hours per week were considered invalid and were excluded for further analysis.

### 2.10 Statistical analysis

Data analysis was performed using the Statistical Products and Service Solutions package version 25 (SPSS Inc., Chicago, IL, U.S.A.). Characteristics of the individuals who responded to the question regarding lifetime prevalence of HE are presented as numbers (n) and proportions (%). Differences between the sexes, and between responders and non‐responders were assessed using chi‐square tests, independent t‐tests, or Mann–Whitney *U* tests. Probability was recognized as significant in where the *P*‐value was < .05. Prevalence is presented as ‘n’ and ‘%’ with corresponding 95% confidence intervals (95%CI). The association between HE in the past year and sex, age, physician diagnosed AD, contact allergy, occupational and non‐occupational wet exposure, overall wet exposure, and work‐related and socioeconomic factors are described using odds ratios (ORs) with corresponding 95%CIs. ORs were calculated by means of univariate and multivariate logistic regression models. In the first multivariate logistic regression model, ORs were adjusted for predefined confounders (age, sex, AD, overall wet exposure, and contact allergy). In the second model ORs were adjusted for all predefined confounders of model 1, plus the socioeconomic and work‐related factors that were statistically significant in the univariate analysis.

The potential presence of effect modification, in which a specific independent variable has a different effect on the main outcome depending on another independent variable, was assessed by including interaction terms in the final model. Interaction terms were defined as all combinations of a specific socioeconomic or work‐related factor multiplied by one of the other socioeconomic or work‐related factors. Effect modification was considered present when *P*‐values of the interaction terms were < .05.

## 3 RESULTS

### 3.1 Study population

Descriptive data regarding potential confounders, socioeconomic, and work‐related factors are presented in Table 1. The lifetime prevalence of HE was 15.0% (95%CI: 14.7‐15.3), and the 1‐year prevalence 7.3% (95%CI: 7.1‐7.5). Females differed statistically significantly from males for almost all variables, including a higher 1‐year and lifetime prevalence of HE, more occupational and non‐occupational wet exposure, lower educational attainment, lower income levels, and differed in employment status compared to males. No significant difference was found for SES.

**TABLE 1:** Characteristics of the study population stratified by sex

	Total, (n = 57 046)	Male, (n = 22 650)	Female, (n = 34 396)	*P*‐value
Hand eczema lifetime prevalence, n (% [95%CI])^a^	8550 (15.0 [14.7‐15.3])	2427 (10.7 [10.3‐11.1])	6123 (17.8 [17.4‐18.2])	**<.001**
Hand eczema lifetime prevalence stratified for age range, n (% [95%CI])^a^				
25‐34 years	528 (17.1 [15.8‐18.5])	93 (12.0 [9.7‐14.3])	435 (18.9 [17.3‐20.5])	**<.001**
35‐44 years	1369 (19.1 [18.2‐20.1])	412 (15.1 [13.7‐16.4])	957 (21.6 [20.4‐22.9])	**<.001**
45‐54 years	2492 (16.9 [16.3‐17.6])	699 (12.7 [11.9‐13.6])	1793 (19.4 [18.6‐20.3])	**<.001**
55‐64 years	2792 (15.2 [14.6‐15.7])	756 (10.5 [9.7‐11.2])	2036 (18.2 [17.5‐18.9])	**<.0**
≥ 65 years	1369 (10.0 [9.5‐10.5])	467 (7.3 [6.6‐7.9])	902 (12.4 [11.7‐13.2])	**<.001**
Hand eczema 1‐year prevalence, n (% [95%CI])^a^	4158 (7.3 [7.1‐7.5])	1228 (5.4 [5.1‐5.7])	2930 (8.4 [8.2‐8.8])	**<.001**
Hand eczema 1‐year prevalence stratified for age range, n (% [95%CI])^a^				
25‐34 years	362 (11.8 [10.6‐12.9])	55 (7.1 [5.3‐8.9])	307 (13.3 [11.9‐14.7])	**<.001**
35‐44 years	821 (11.5 [10.7‐12.2])	254 (9.3 [8.2‐10.4])	567 (12.8 [11.8‐13.8])	**<.001**
45‐54 years	1240 (8.4 [8.0‐8.9])	256 (4.7 [4.1‐5.2])	884 (9.6 [8.9‐10.2])	**<.001**
55‐64 years	1219 (6.6 [6.3‐7.0])	369 (5.1 [4.6‐5.6])	850 (7.6 [7.1‐8.1])	**<.001**
≥ 65 years	516 (3.8 [3.5‐4.1])	194 (3.0 [2.6‐3.4])	322 (4.4 [4.0‐4.9])	**<.001**
Age in years, mean (± SD)^b^	55.8 (12.2)	57.3 (12.2)	54.7 (12.1)	**<.001**
Age range^b^				
25‐34 years	3080 (5.4)	773 (3.4)	2307 (6.7)	**<.001**
35‐44 years	7151 (12.5)	2730 (12.1)	4421 (12.9)	**.005**
45‐54 years	14 704 (25.8)	5485 (24.2)	9219 (26.8)	**<.001**
55‐64 years	18 422 (32.3)	7231 (31.9)	11 191 (32.5)	0.13
≥ 65 years	13 689 (24.0)	6431 (28.4)	7258 (21.1)	**<.001**
Sex^b^ n (%)				
Male	22 650 (39.7)	22 650 (100.0)	‐	‐
Female	34 396 (60.3)	‐	34 396 (100.0)	‐
Atopic dermatitis^a^ ^,^ ^c^ n (%)	5145 (9.2)	1457 (6.5)	3688 (10.9)	**<.001**
Patch test^a^ n (%)				
Yes	9461 (16.6)	2811 (12.4)	6650 (19.4)	**<.001**
Contact allergy^a^ ^,^ ^d^ n (%)				
No	2336 (24.8)	887 (31.7)	1449 (21.9)	
Yes	7070 (75.2)	1914 (68.3)	5156 (78.1)	**<.001**
Overall wet exposure^a^ n (%)	13 299 (24.6)	2724 (12.6)	10 575 (32.6)	**<.001**
Occupational wet exposure^a^ n (%)	8760 (16.1)	1774 (8.1)	6986 (21.3)	**<.001**
Direct contact with water, fluids and/or moist products at work (hours/day)^a^ n (%)				
Never	13 033 (23.7)	5115 (23.3)	7918 (24.0)	.08
< 0.5	24 961 (45.4)	11 987 (54.6)	12 974 (39.2)	**<.001**
0.5‐1	8297 (15.1)	2917 (13.3)	5380 (16.3)	**<.001**
1‐2	4567 (8.3)	1128 (5.1)	3439 (10.4)	**<.001**
> 2	4146 (7.5)	798 (3.6)	3348 (10.1)	**<.001**
Wearing gloves at work (hours/day)^a^ n (%)				
Never	43 054 (78.1)	18 290 (83.2)	24 764 (74.8)	**<.001**
< 0.5	5146 (9.3)	2160 (9.8)	2986 (9.0)	**.001**
0‐5‐1	2345 (4.3)	588 (2.7)	1757 (5.3)	**<.001**
1‐2	1845 (3.3)	591 (2.7)	2127 (6.4)	**<.001**
> 2	2718 (4.9)	362 (1.6)	1483 (4.5)	**<.001**
Frequency of hand washing at work per day^a^ n (%)				
Never	10 543 (19.2)	3965 (18.1)	6578 (19.9)	**<.001**
< 5	17 710 (32.2)	8767 (39.9)	8943 (27.1)	**<.001**
5‐10	17 255 (31.4)	7361 (33.5)	9894 (29.9)	**<.001**
10‐20	6227 (11.3)	1444 (6.6)	4783 (14.5)	**<.001**
> 20	3262 (5.9)	416 (1.9)	2846 (8.6)	**<.001**
Non‐occupational wet exposure^a^ n (%)	5626 (10.0)	823 (3.7)	4803 (14.1)	**<.001**
Direct contact with water, fluids and/or moist products at home (hours/day)^a^ n (%)				
Never	688 (1.2)	366 (1.6)	322 (0.9)	**<.001**
< 0,5	24 348 (42.9)	13 549 (60.2)	10 799 (31.6)	**<.001**
0,5‐1	21 980 (38.8)	6932 (30.8)	15 048 (44.0)	**<.001**
1‐2	7562 (13.3)	1284 (5.7)	6278 (18.4)	**<.001**
> 2	2123 (3.7)	385 (1.7)	1738 (5.1)	**<.001**
Wearing gloves at home (hours/day)^a^ n (%)				
Never	47 938 (84.5)	20 127 (89.4)	27 811 (81.3)	**<.001**
< 0.5	7428 (13.1)	2156 (9.6)	5272 (15.4)	**<.001**
0,5‐1	961 (1.7)	167 (0.7)	794 (2.3)	**<.001**
1‐2	247 (0.4)	40 (0.2)	207 (0.6)	**<.001**
> 2	140 (0.2)	25 (0.1)	115 (0.3)	**<.001**
Frequency of hand washing at home per day^a^ n (%)				
Never	217 (0.4)	111 (0.5)	106 (0.3)	**.001**
< 5	18 362 (32.4)	10 202 (45.3)	8160 (23.9)	**<.001**
5‐10	28 609 (50.4)	10 449 (46.4)	18 160 (53.1)	**<.001**
10‐20	8388 (14.8)	1616 (7.2)	6772 (19.8)	**<.001**
> 20	1155 (2.0)	146 (0.6)	1009 (2.9)	**<.001**
Educational attainment^b^ n (%)				
Low	14 873 (26.5)	5868 (26.4)	9005 (26.6)	0.52
Middle	21 969 (39.2)	8160 (36.7)	13 809 (40.8)	**<.001**
High	19 211 (34.3)	8210 (36.9)	11 001 (32.5)	**<.001**
SES^b^ n (%)				
Low	15 204 (34.8)	5927 (34.3)	9277 (35.2)	.05
Middle	25 869 (59.2)	10 316 (56.9)	15 553 (59.0)	.15
High	2604 (6.0)	1053 (6.1)	1551 (5.9)	.37
Nett household income (euros per month)^b^ n (%)				
< 750	1749 (3.6)	396 (2.0)	1353 (4.8)	**<.001**
750‐1000	1299 (2.7)	301 (1.5)	998 (3.5)	**<.001**
1000‐1500	3951 (8.2)	1079 (5.5)	2872 (10.1)	**<.001**
1500‐2000	6844 (14.2)	2846 (14.4)	3998 (14.1)	.24
2000‐2500	7984 (16.6)	3555 (18.0)	4429 (15.6)	**<.001**
2500‐3000	8593 (17.8)	3713 (18.8)	4880 (17.2)	**<.001**
3000‐3500	7634 (15.9)	3204 (16.3)	4430 (15.6)	**.046**
> 3500	10 088 (21.0)	4615 (23.4)	5473 (19.2)	**<.001**
Employment status^b^ n (%)				
Unemployed	5672 (10.3)	1055 (4.8)	4617 (13.8)	**<.001**
Retired	4232 (7.7)	2444 (11.2)	1788 (5.4)	**<.001**
Unfit for work	1186 (2.1)	441 (2.0)	745 (2.2)	.09
Employed	44 177 (79.9)	17 929 (82.0)	26 248 (78.6)	**<.001**
Workhours per week, mean (± SD)^b^	30.3 (13.1)	38.5 (11.6)	24.8 (11.1)	**<.001**
Workhours (hours/week),^b^ n (%)				
1‐8	3075 (6.7)	705 (3.8)	2370 (8.7)	**<.001**
9‐16	4473 (9.8)	512 (2.8)	3961 (14.5)	**<.001**
17‐24	8315 (18.2)	511 (2.8)	7804 (28.6)	**<.001**
25‐32	9103 (19.9)	1769 (9.6)	7334 (26.9)	**<.001**
33‐40	14 750 (32.3)	9999 (54.2)	4751 (17.4)	**<.001**
> 40	5986 (13.1)	4951 (26.8)	1035 (3.8)	**<.001**
ISCO‐08 major occupation groups^b^ ^,^ n (%)				
Managers	96 (0.2)	72 (0.3)	24 (0.1)	**<.001**
Professionals	2844 (5.2)	2011 (9.2)	833 (2.5)	**<.001**
Technicians and associate professionals	14 129 (25.7)	6076 (27.9)	8053 (24.3)	**<.001**
Clerical support workers	10 739 (19.6)	3961 (18.2)	6778 (20.5)	**<.001**
Service and sales workers	7113 (13.0)	1811 (8.3)	5302 (16.0)	**<.001**
Skilled agricultural, forestry and fishery workers	10 969 (20.0)	2128 (9.8)	8841 (26.7)	**<.001**
Craft and related trades workers	1209 (2.2)	875 (4.0)	334 (1.0)	**<.001**
Plants and machine operators and assemblers	3373 (6.1)	2895 (13.3)	478 (1.4)	**<.001**
Elementary occupations	1539 (2.8)	1215 (5.6)	324 (1.0)	**<.001**
Armed forces occupations	2888 (5.3)	724 (3.3)	2164 (6.5)	**<.001**

*Note*: *P*‐values <0.05 are shown in bold.

*Note*: Wet exposure was defined as: 1, activities where individuals have to immerse their hands in liquids for >2 hours daily, or wear (occlusive) gloves for > 2 hours daily, or wash their hands >20 times a day; or 2, a combination of two of the following: activities where individuals have to immerse their hands in liquids for 1‐2 hours daily, wear (occlusive) gloves for 1‐2 hours daily, or wash their hands 10‐20 times a day (see also Appendix S1).

Abbreviations: CI, confidence interval; *ISCO‐08*, International Standard Classification of Occupations 2008; SD, Standard deviation; SES, socioeconomic status; n, number. For data on missing values see Appendix S3.

^a^ Characteristics evaluated during baseline assessment (2006‐2013).

^b^ Characteristics included in the add‐on study, sent out in 2020.

^c^ Self‐reported physician diagnosed atopic dermatitis .

^d^ Percentages based on total of participants ever being patch tested and reporting at least one positive reaction.

In the non‐responder analysis, there was a statistically significantly increased proportion of female responders compared to male responders. Responders were also more likely to be older, higher educated, with a higher SES, higher income, a tendency to report psoriasis and eczema more often, and differed in employment status (Appendix S2).

### 3.2 Predefined confounders

The results of the logistic regression analyses to assess different risk factors for HE are shown in Table 2. The univariate analysis showed a positive association between HE in the past year and female sex (OR 1.71, [95%CI: 1.59‐1.83]), AD (OR 8.40, [95%CI: 7.78‐9.07]), contact allergy (OR 1.86 [95%CI: 1.60‐2.16]) and overall wet exposure (OR 1.64 [95%CI: 1.53‐1.75]. A negative association was found for higher age groups (45‐54 years: OR 0.72 [95%CI: 0.63‐0.81]; 55‐65 years: OR 0.55 [95%CI: 0.49‐0.62]; ≥ 65 years: OR 0.30 [95%CI: 0.26‐0.34]). After adjustment for all predefined confounders (age, sex, AD, contact allergy, and overall wet exposure) (model 1), and when adjusting for the predefined confounders and all other variables which were statistically significant in the univariate analysis (model 2), similar results were found regarding the predefined confounders.

**TABLE 2:** Univariate and multivariate logistic regression analysis for the association between reporting hand eczema in the past year and wet exposure, socioeconomic, and occupational factors

	1‐year prevalence of hand eczema	No hand eczema (lifetime),	Crude OR (95%CI)	*P*‐value	Adjusted OR^e^ (95%CI) model 1	*P*‐value	Adjusted OR^f^ (95%CI) model 2	*P*‐value
	n (%)	n (%)						
Total	4158 (7.3)	48 496 (85.0)	‐	‐	‐	‐	‐	‐
Age range^b^								
25‐34 years	362 (8.7)	2552 (5.3)	1	‐	1	‐	1	**‐**
35‐44 years	821 (19.7)	5782 (11.9)	1.00 (0.88‐1.14)	0.99	1.08 (0.93‐1.24)	0.33	1.10 (0.90‐1.33)	.35
45‐54 years	1240 (29.8)	12 212 (25.2)	**0.72 (0.63‐0.81)**	**<0.001**	**0.75 (0.65‐0.86)**	**<0.001**	**0.78 (0.64‐0.94)**	**.01**
55‐64 years	1219 (29.3)	15 630 (32.2)	**0.55 (0.49‐0.62)**	**<0.001**	**0.61 (0.53‐0.70)**	**<0.001**	**0.67 (0.55‐0.81)**	**<.001**
≥ 65 years	516 (12.4)	12 320 (25.4)	**0.30 (0.26‐0.34)**	**<0.001**	**0.36 (0.31‐0.43)**	**<0.001**	**0.46 (0.36‐0.58)**	**<.001**
Sex^b^								
Male	1228 (29.5)	20 223 (41.7)	1	‐	1	**‐**	1	**‐**
Female	2930 (70.5)	28 273 (58.3)	**1.71 (1.59‐1.83)**	**<0.001**	**1.27 (1.18‐1.38)**	**<0.001**	**1.20 (1.08‐1.34)**	**.001**
Atopic dermatitis^a^ ^,^ ^c^								
No	2628 (66.3)	45 177 (94.3)	1	‐	1	**‐**	1	**‐**
Yes	1334 (33.7)	2730 (5.7)	**8.40 (7.78‐9.07)**	**<0.001**	**5.89 (5.42‐6.40)**	**<0.001**	**5.97 (5.42‐6.58)**	**<.001**
Patch test^a^								
No	2800 (67.4)	41 878 (86.5)	‐	‐	‐	**‐**	**‐**	**‐**
Yes	1352 (32.6)	6551 (13.5)	‐	‐	‐	**‐**	**‐**	**‐**
Contact allergy^a^ ^,^ ^d^								
No	232 (17.2)	1813 (27.8)	1	‐	1	**‐**	1	**‐**
Yes	1119 (82.8)	4704 (72.2)	**1.86 (1.60‐2.16)**	**<0.001**	**1.36 (1.15‐1.61)**	**<0.001**	**1.26 (1.04‐1.53)**	**.02**
Overall wet exposure^a^								
No	2651 (66.9)	35 394 (76.8)	1	‐	1	‐	1	**‐**
Yes	1309 (33.1)	10 689 (23.2)	**1.64 (1.53‐1.75)**	**<0.001**	**1.33 (1.23‐1.43)**	**<0.001**	**1.29 (1.17‐1.42)**	**<.001**
Occupational wet exposure^a^								
No	3076 (76.8)	39 492 (85.1)	1	‐	1	**‐**	1	‐
Yes	931 (23.2)	6892 (14.9)	**1.73 (1.61‐1.87)**	**<0.001**	**1.36 (1.25‐1.48)**	**<0.001**	**1.35 (1.22‐1.49)**	**<.001**
Direct contact with water, fluids and/or moist products at work (hours/day)^a^								
< 0.5	2494 (61.7)	32 924 (70.4)	1	‐	1	‐	1	‐
0.5‐1	691 (17.1)	6926 (14.8)	**1.32 (1.21‐1.44)**	**<0.001**	**1.12 (1.02‐1.24)**	**0.02**	**1.14 (1.02‐1.27)**	**.02**
1‐2	387 (9.6)	3732 (8.0)	**1.37 (1.23‐1.53)**	**<0.001**	1.09 (0.96‐1.23)	0.19	1.06 (0.92‐1.23)	.42
> 2	467 (11.6)	3192 (6.8)	**1.93 (1.74‐2.15)**	**<0.001**	**1.48 (1.31‐1.66)**	**<0.001**	**1.52 (1.32‐1.75)**	**<.001**
Wearing gloves at work (hours/day)^a^								
< 0.5	3269 (80.7)	41 443 (88.5)	1	‐	1	‐	1	‐
0.5‐1	266 (6.6)	1842 (3.9)	**1.83 (1.60‐2.09)**	**<0.001**	**1.48 (1.28‐1.72)**	**<0.001**	**1.42 (1.19‐1.68)**	**<.001**
1‐2	195 (4.8)	1439 (3.1)	**1.72 (1.47‐2.00)**	**<0.001**	**1.36 (1.15‐1.61)**	**<0.001**	**1.27 (1.05‐1.55)**	**.02**
> 2	321 (7.9)	2121 (4.5)	**1.92 (1.70‐2.17)**	**<0.001**	**1.48 (1.29‐1.69)**	**<0.001**	**1.53 (1.31‐1.78)**	**<.001**
Frequency of hand washing at work per day^a^								
< 5	1721 (42.6)	24 631 (52.7)	1	‐	1	‐	1	‐
5‐10	1327 (32.9)	14 566 (31.1)	**1.30 (1.21‐1.41)**	**<0.001**	**1.09 (1.01‐1.19)**	**0.04**	**1.16 (1.05‐1.27)**	**.002**
10‐20	599 (14.8)	5018 (10.7)	**1.71 (1.55‐1.88)**	**<0.001**	**1.23 (1.10‐1.37)**	**<0.001**	**1.29 (1.14‐1.47)**	**<.001**
> 20	391 (9.7)	2547 (5.4)	**2.20 (1.95‐2.47)**	**<0.001**	**1.54 (1.35‐1.76)**	**<0.001**	**1.54 (1.32‐1.80)**	**<.001**
Non‐occupational wet exposure^a^								
No	3519 (86.0)	43 437 (90.5)	1	‐	1	‐	1	‐
Yes	575 (14.0)	4561 (9.5)	**1.56 (1.42‐1.71)**	**<0.001**	**1.42 (1.28‐1.58)**	**<0.001**	**1.34 (1.17‐1.53)**	**<.001**
Direct contact with water, fluids and/or moist products at home (hours/day)^a^								
< 0.5	1731 (42.0)	21 443 (44.4)	1	‐	1	‐	1	‐
0.5‐1	1559 (37.8)	18 764 (38.9)	1.03 (0.96‐1.11)	0.43	0.98 (0.90‐1.06)	0.58	0.98 (0.89‐1.07)	.58
1‐2	627 (15.2)	6313 (13.1)	**1.23 (1.12‐1.35)**	**<0.001**	**1.12 (1.00‐1.25)**	**0.04**	1.05 (0.92‐1.20)	.46
> 2	208 (5.0)	1723 (3.6)	**1.50 (1.29‐1.74)**	**<0.001**	**1.28 (1.08‐1.52)**	**0.004**	1.24 (1.00‐1.53)	.05
Wearing gloves at home (hours/day)^a^								
< 0.5	3879 (93.9)	47 313 (98.1)	1	‐	1	‐	1	‐
0.5‐1	157 (3.8)	683 (1.4)	**2.80 (2.35‐3.35)**	**<0.001**	**2.57 (2.10‐3.15)**	**<0.001**	**2.31 (1.76‐3.04)**	**<.001**
1‐2	56 (1.4)	161 (0.3)	**4.24 (3.13‐5.76)**	**<0.001**	**3.59 (2.52‐5.09)**	**<0.001**	**3.22 (2.00‐5.17)**	**<.001**
> 2	39 (0.9)	90 (0.2)	**5.29 (3.63‐7.71)**	**<0.001**	**3.61 (2.33‐5.60)**	**<0.001**	**3.53 (2.00‐6.22)**	**<.001**
Frequency of hand washing at home per day^a^								
< 5	1255 (30.4)	15 932 (33.0)	1	‐	1	‐	1	‐
5‐10	1954 (47.4)	24 470 (50.7)	1.01 (0.94‐1.09)	0.72	1.01 (0.93‐1.10)	0.77	1.04 (0.95‐1.14)	.44
10‐20	784 (19.0)	6926 (14.3)	**1.44 (1.31‐1.58)**	**<0.001**	**1.34 (1.21‐1.49)**	**<0.001**	**1.30 (1.15‐1.47)**	**<.001**
> 20	131 (3.2)	944 (2.0)	**1.76 (1.46‐2.13)**	**<0.001**	**1.62 (1.31‐2.00)**	**<0.001**	**1.58 (1.20‐2.09)**	**.001**
Educational attainment^b^								
Low	840 (20.5)	12 959 (27.2)	1	**‐**	1	‐	1	**‐**
Middle	1792 (43.7)	18 370 (38.6)	**1.51 (1.38‐1.64)**	**<0.001**	**1.19 (1.08‐1.32)**	**<0.001**	**1.17 (1.04‐1.32)**	**.01**
High	1468 (35.8)	16 307 (34.2)	**1.39 (1.27‐1.52)**	**<0.001**	**1.17 (1.06‐1.30)**	**0.002**	**1.18 (1.04‐1.34)**	**.01**
SES^b^								
Low	1135 (35.3)	12 863 (34.7)	1	‐	1	‐	1	‐
Middle	1882 (58.6)	22 016 (59.4)	0.97 (0.90‐1.05)	0.42	1.03 (0.94‐1.12)	0.55	1.05 (0.95‐1.15)	.39
High	195 (6.1)	2207 (6.0)	1.00 (0.86‐1.17)	0.99	1.04 (0.87‐1.24)	0.66	1.02 (0.83‐1.25)	.86
Nett household income (euros per month)^b^								
< 750	185 (5.2)	1456 (3.6)	1	‐	1	‐	1	‐
750‐1000	129 (3.6)	1072 (2.6)	0.95 (0.75‐1.20)	0.66	1.21 (0.92‐1.6)	0.17	1.19 (0.85‐1.67)	.31
1000‐1500	334 (9.3)	3299 (8.1)	**0.80 (0.66‐0.96)**	**0.02**	1.12 (0.89‐1.42)	0.33	1.07 (0.81‐1.42)	.64
1500‐2000	504 (14.1)	5833 (14.3)	**0.68 (0.57‐0.81)**	**<0.001**	1.05 (0.83‐1.31)	0.71	1.01 (0.76‐1.32)	.97
2000‐2500	607 (17.0)	6751 (16.5)	**0.71 (0.60‐0.84)**	**<0.001**	1.13 (0.90‐1.42)	0.29	1.10 (0.84‐1.44)	.5
2500‐3000	611 (17.1)	7261 (17.8)	**0.66 (0.56‐0.79)**	**<0.001**	1.05 (0.84‐1.31)	0.69	1.02 (0.78‐1.33)	.91
3000‐3500	549 (15.3)	6489 (15.9)	**0.67 (0.56‐0.79)**	**<0.001**	1.02 (0.81‐1.28)	0.89	0.95 (0.72‐1.25)	.7
> 3500	660 (18.4)	8701 (21.3)	**0.60 (0.50‐0.71)**	**<0.001**	0.99 (0.79‐1.24)	0.93	0.92 (0.70‐1.21)	0.56
Employment status^b^								
Unemployed	435 (10.7)	4772 (10.2)	1	‐	1	‐	1	‐
Retired	122 (3.0)	3904 (8.3)	**0.34 (0.28‐0.42)**	**<0.001**	**0.69 (0.53‐0.90)**	**0.01**	0.37 (0.05‐2.82)	.34
Unfit for work	98 (2.4)	978 (2.1)	1.10 (0.87‐1.38)	0.43	1.03 (0.79‐1.36)	0.81	0.34 (0.04‐2.56)	.29
Employed	3398 (83.8)	37 295 (79.4)	1.00 (0.90‐1.11)	0.98	0.89 (0.79‐1.01)	0.08	1.13 (0.75‐1.69)	.56
Workhours per week, mean +/‐ SD^b^	29.2 **+/‐** 12.6	30.6 **+/‐** 13.2	**0.86 (0.82‐0.89)**	**<0.001**	1.01 (0.995‐1.002)	0.53	1.00 (0.99‐1.00)	.36
Workhours (hours/week)^b^								
1‐8	226 (6.5)	2610 (6.8)	1	‐	1	‐	1	‐
9‐16	387 (11.1)	3662 (9.5)	**1.22 (1.03‐1.45)**	**0.02**	1.10 (0.90‐1.33)	0.36	1.09 (0.87‐1.37)	.47
17‐24	709 (20.3)	6845 (17.7)	**1.20 (1.02‐1.40)**	**0.03**	1.06 (0.89‐1.27)	0.51	1.06 (0.85‐1.33)	.59
25‐32	772 (22.1)	7551 (19.6)	**1.18 (1.01‐1.38)**	**0.04**	1.08 (0.91‐1.29)	0.4	1.08 (0.86‐1.34)	.51
33‐40	1010 (28.9)	12 746 (33.0)	0.92 (0.79‐1.06)	0.25	1.04 (0.87‐1.24)	0.7	1.00 (0.80‐1.24)	.97
> 40	394 (11.3)	5188 (13.4)	0.88 (0.74‐1.04)	0.13	1.08 (0.88‐1.32)	0.45	1.08 (0.84‐1.38)	.55

*Note*: Statistically significant odds ratios are shown in bold.

*Note*: Wet exposure was defined as: 1. activities where individuals have to immerse their hands in liquids for >2 hours daily, or wear (occlusive) gloves for a > 2 hours daily, or wash their hands >20 times a day; or 2. a combination of two of the following: activities where individuals have to immerse their hands in liquids for 1‐2 hours daily, wear (occlusive) gloves for 1‐2 hours daily, or wash their hands 10‐20 times a day (see Appendix S1).

Abbreviations: CI, confidence interval; SES, socioeconomic status; n, number; OR, odds ratio. For data on missing values see Appendix S3.

^a^ Model 1: Binary logistic regression analysis with the outcome OR of the 1‐year prevalence of hand eczema, adjusted for sex, age, atopic dermatitis, contact allergy, and overall exposure to wet work. For occupational and non‐occupational wet work exposure as well as for the specific components contributing to exposure at work and at home no adjustment was made for overall exposure to wet work in the corresponding logistic regression models.

^b^ Model 2: Binary logistic regression analysis with the outcome OR of the 1‐year prevalence of hand eczema, adjusted for sex, age, atopic dermatitis, contact allergy, overall exposure to wet work, and educational attainment, employment status, income and workhours. For occupational and non‐occupational wet work exposure as well as for the specific components contributing to exposure at work and at home no adjustment was made for overall exposure to wet work in the corresponding logistic regression models.

^c^ Characteristics evaluated during baseline assessment (2006‐2013).

^d^ Characteristics included in the add‐on study, sent out in 2020.

^e^ Self‐reported physician diagnosed atopic dermatitis.

^f^ Percentages based on the total of participants ever being patch tested and reporting at least one positive reaction.

### 3.3 Occupational and non‐occupational wet exposure

The univariate analysis showed a positive association between HE in the past year and both occupational wet exposure and non‐occupational wet exposure (OR 1.73 [95%CI: 1.61‐1.87] and OR 1.56 [95%CI: 1.42‐1.71], respectively). After adjustment (model 1 and 2) a positive association with HE remained (model 2: OR 1.35 [95%CI: 1.22‐1.49] and OR 1.34 [95%CI: 1.17‐1.53], respectively).

When looking further into the specific factors contributing to wet exposure, contact with fluids, use of gloves and frequency of hand washing contributed to occupational wet exposure, and use of gloves and frequency of hand washing contributed to non‐occupational wet exposure.

### 3.4 Occupational groups

Table 3 shows the ISCO‐08 occupational groups with at least 75 cases of HE in the past year, including the crude prevalence and the corresponding ORs adjusted for age and sex. The highest 1‐year prevalence rates of HE were found in the occupations classified as personal care workers in health services (7.4%), legal, social and religious associate professionals (5.1%), shop salespersons (4.9%), nursing and midwifery professionals (4.2%) and domestic, hotel and office cleaners and helpers (3.7%). Corresponding unadjusted ORs showed a positive association between HE in the past year and all the above‐mentioned occupations, except for shop salespersons (OR 1.33 [95%CI: 1.17‐1.50]; OR 1.42 [95%CI 1.22‐1.65]; OR 1.03 [95%CI: 0.89‐1.20]; OR 1.47 [95%CI: 1.25‐1.74]; and OR 1.24 [95%CI: 1.05‐1.48], respectively). After adjusting for age and sex, a positive association was found between HE in the past year and the occupational groups nursing and midwifery professionals (OR 1.25 [95%CI: 1.06‐1.48]), legal, social and religious associate professionals (OR 1.19 [95%CI: 1.03‐1.39]), and personal care workers in health services (OR 1.20 [95%CI: 1.06‐1.36]). Table 4 shows the 1‐year prevalence of HE while working in high‐risk occupations during the onset of HE. The highest proportion of participants with HE in the past year reported onset of HE while working as healthcare workers (17.9%), housekeepers and cleaners (7.7%), and agricultural workers (2.9%).

**TABLE 3:** The frequency of hand eczema in the past year, analyzed in major occupational groups (classified on the 3‐digit level of the ISCO‐08 classification) with at least 75 cases of hand eczema in the past year

ISCO‐08	Occupational group	Total n (%)	1‐year prevalence of hand eczema n (%)	No hand eczema (lifetime) N (%) n = 48 496	Crude OR (95%CI)	*P*‐value	Adjusted OR^a^ (95%CI)	*P*‐value
5320	Personal care workers in health services	3305 (6.0)	296 (7.4)	2645 (5.7)	**1.33 (1.17‐1.50)**	**<0.001**	**1.20 (1.06‐1.36)**	**.005**
3410	Legal, social and religious associate professionals	2064 (3.8)	203 (5.1)	1689 (3.6)	**1.42 (1.22‐1.65)**	**<0.001**	**1.19 (1.03‐1.39)**	**.02**
5220	Shop salespersons	2619 (4.8)	197 (4.9)	2220 (4.8)	1.03 (0.89‐1.20)	0.67	0.92 (0.79‐1.07)	.26
2220	Nursing and midwifery professionals	1679 (3.1)	167 (4.2)	1335 (2.9)	**1.47 (1.25‐1.74)**	**<0.001**	**1.25 (1.06‐1.48)**	**.008**
9110	Domestic, hotel and office cleaners and helpers	1720 (3.1)	150 (3.7)	1414 (3.0)	**1.24 (1.05‐1.48)**	**0.01**	1.17 (0.98‐1.39)	.08
2340	Primary school and early childhood teachers	1531 (2.8)	116 (2.9)	1291 (2.8)	1.05 (0.86‐1.27)	0.65	0.95 (0.78‐1.16)	.62
4310	Numerical clerks	1398 (2.5)	113 (2.8)	1185 (2.5)	1.11 (0.91‐1.35)	0.29	1.04 (0.86‐1.27)	.67
4220	Client information workers	1392 (2.5)	111 (2.8)	1175 (2.5)	1.10 (0.90‐1.34)	0.34	0.98 (0.80‐1.19)	.83
2420	Administration professionals	1538 (2.8)	107 (2.7)	1315 (2.8)	0.95 (0.77‐1.15)	0.58	0.99 (0.81‐1.21)	.90
3340	Administrative and specialized secretaries	1243 (2.3)	95 (2.4)	1034 (2.2)	1.070 (0.87‐1.32)	0.533	0.95 (0.77‐1.18)	.62
5310	Child‐care workers and teachers' aides	1120 (2.0)	94 (2.3)	917 (2.0)	1.20 (0.97‐1.48)	0.10	1.07 (0.86‐1.33)	.52
3250	Other health associate professionals	1234 (2.2)	88 (2.2)	1020 (2.2)	1.00 (0.81‐1.25)	0.98	0.85 (0.68‐1.06)	.14
4410	Other clerical support workers	1146 (2.1)	86 (2.1)	983 (2.1)	1.02 (0.81‐1.27)	0.88	0.99 (0.79‐1.24)	.92
2630	Social and religious professionals	892 (1.6)	75 (1.9)	758 (1.6)	1.15 (0.91‐1.47)	0.24	1.01 (0.79‐1.29)	.92

*Note*: Statistically significant odds ratios are shown in bold.

Abbreviations: *ISCO‐08*, International Standard Classification of Occupations 2008; *n*, number; *OR* Odds ratio; *CI*, confidence interval. For data on missing values see online supplement S3.

^a^ Binary logistic regression analysis with the outcome OR of the 1‐year prevalence of hand eczema, adjusted for sex and age.

**TABLE 4:** Proportion of participants reporting onset of hand eczema while working in high‐risk occupations

High‐risk occupation	1‐year prevalence of hand eczema, (n = 4158) n (%)
Agricultural workers/gardeners	122 (2.9)
Bakers/pastry makers	23 (0.6)
Beauty specialists/nail stylists	24 (0.6)
Butchers/slaughterhouse workers	19 (0.5)
Canning and fish processing industry workers	<10 (<0.2)
Food industry	63 (1.5)
Construction workers/carpenters	75 (1.8)
Cooks/kitchen workers/vegetable processers	71 (1.7)
Dental technicians	17 (0.4)
Fitters	102 (2.5)
Florists	18 (0.4)
Hairdressers	39 (0.9)
Health‐care workers	744 (17.9)
Housekeepers/cleaners	320 (7.7)
Metal surface processers	54 (1.3)
Painters and varnishers	25 (0.6)
Plasterers	<10 (<0.2)
Tile setters and terazzo workers	<10 (<0.2)
Print or paper industry	18 (0.4)
Textile, leather, fur or pelts industry	12 (0.3)

Abbreviations: *n*, number. For data on missing values see Appendix S3.

### 3.5 Socioeconomic factors

Regarding socioeconomic factors, the univariate analysis (Table 2) showed a positive association between HE in the past year and higher levels of educational attainment (OR 1.51 [95%CI: 1.38‐1.64] and OR 1.39 [95%CI: 1.27‐1.52], for middle and high educational attainment, respectively). A negative association was found for higher income categories (ORs varying from 0.60 [95%CI: 0.50‐0.71]) for the highest income category and OR 0.95 [95%CI: 0.75‐1.20] for one of the lowest income categories), being retired (OR 0.34 [95%CI: 0.28‐0.42]), and number of working hours per week (OR 0.86 [95%CI: 0.82‐0.89]). In the first model, a positive association with HE remained for higher levels of educational attainment (OR 1.19 [95%CI: 1.08‐1.32] and OR 1.17 [95%CI: 1.06‐1.30] for middle and high educational attainment, respectively) and a negative association remained for being retired (OR 0.69 [95%CI: 0.53‐0.90]). In the second model, a positive association for higher levels of educational attainment remained (OR 1.17 [95%CI: 1.04‐1.32] and OR 1.18 [95%CI: 1.04‐1.34] for middle and high educational attainment, respectively), but no statistically significant association remained for being retired (OR 0.37 [95%CI: 0.05‐2.82]). The univariate analyses, as well as both adjusted models, showed no significant association between SES and HE in the past year (model 2: OR 1.05 [95%CI: 0.95‐1.15] and OR 1.02 [95%CI: 0.83‐1.25] for middle and high SES, respectively). When adding interaction terms to the second model, no effect modification was found.

## 4 DISCUSSION

The main findings of this study were a positive association between HE in the past year and occupational as well as non‐occupational wet exposure. Specific factors contributing to occupational wet exposure included direct contact with fluids, use of gloves, and frequency of hand washing. Specific factors for non‐occupational wet exposure included use of gloves and frequency of hand washing. Apart from a positive association between HE and certain high‐risk occupations, a positive association was found for occupations not considered as high‐risk occupations as well. In addition, a positive association between reporting HE in the past year and higher levels of education was found. No association was found for SES, income, employment status, or number of working hours.

### 4.1 Occupational and non‐occupational wet exposure

The association between HE and wet work exposure has been studied previously. However, most studies only focus on occupational exposure and not on non‐occupational exposure.^7^, ^8^, ^9^, ^10^, ^11^ In line with our study, a study among healthcare workers in Sweden, found that frequent hand washing and wearing gloves at home as well as at work were associated with HE.^8^ In another cross‐sectional questionnaire‐based study among healthcare workers, a dose‐dependent association was found between self‐reported HE in the past year and occupational hand washing with soap as well as with time spent wearing disposable non‐sterile gloves at the workplace. In contrast to the current study, hand washing with soap at home showed no association with HE.^9^ However, as the study population differed (healthcare workers vs. general population) and the questions in the current study did not specify the use of soap, results are difficult to compare. Overall, the current study has shown that overall non‐occupational exposure should be considered as an additional risk factor for HE in the Dutch general population. Therefore, exposure during the entire day needs to be considered in the prevention of HE, as well as in counselling patients with HE.

### 4.2 Occupations

In the current study, a higher 1‐year prevalence of HE among healthcare workers, including personal care workers and nursing and midwifery professionals was found. Previous studies have shown that healthcare workers are at an increased risk of developing HE.^5^, ^13^, ^35^ As wet work is shown to be a major risk factor for HE, it is likely that working in the healthcare sector, which generally includes a significant amount of wet work exposure, is associated with reporting HE. However, the current study found no higher 1‐year prevalence of HE in the occupational group compromising other health associate professionals. This discrepancy could be due to differences in exposure, as tasks of associate professionals may differ from tasks of nursing and midwifery professionals. This discrepancy was found as well in a previous study among clinical patients with irritant contact dermatitis.^36^ A positive association between reporting HE in the past year and legal, social and religious associate professionals was found as well. This occupational group does include a wide range of occupations that are not typically considered as high‐risk occupations. However, frequent hand washing or exposure to for example food products could be part of professions such as a sheltered housing supervisor or personal supervisor, which are examples of occupations included in the legal, social and religious associate occupational group. Tasks may, for example, include supervision of vulnerable persons living in sheltered home services. As described previously, when analyzing the association between high‐risk occupations and HE, a “healthy worker effect” should be taken into account.^14^, ^37^ This phenomenon, in which individuals with HE might avoid jobs, switch jobs after development of HE, or might be advised not to choose risk occupations, could lead to a tendency of equalization of HE prevalence between high‐risk and low‐risk occupations. Overall, the classification into high‐risk and low‐risk occupations does not always reflect the true risk of HE. Job tasks and, therefore, exposure to irritants could vary broadly between occupational groups. Moreover, previous studies found challenging differences in exposure to irritants within occupational groups, with women and younger people reporting more exposure to water.^38^, ^39^ Therefore, to precisely determine whether an individual is working in a high‐risk occupation, the job tasks and level of exposure to irritants are of more importance than the occupational group or job title alone.

### 4.3 Socioeconomic factors

The association between HE and socioeconomic factors, such as income and educational attainment, has been studied previously.^15^, ^16^, ^17^, ^19^ A Swedish nationwide survey found that 1‐year prevalence of HE was significantly higher for individuals within the highest household income category, compared with those within the lowest income category, whereas another cross‐sectional study among the general population of Oslo found that self‐reported current HE was significantly higher for individuals with middle household income compared to low household income, while no association with high household income was found.^16^, ^19^ However, different income categories were studied, which makes it difficult to compare these findings. In a cohort study among young adults in the general population and also in the Swedish nationwide survey study, no association between educational attainment and HE in the past year was found.^17^, ^19^ Furthermore, in a population‐based, twin cohort study and in a register‐based cohort study among individuals with HE no significant association between the prognosis of HE and level of education was found.^15^ On the other hand, Dalgard et al. found that in the general population individuals with lower education reported significantly more HE than those with the highest level of education.^16^ Data from a survey in the general population conducted in five European countries showed that people with high SES reported a higher lifetime prevalence of skin diseases, including contact dermatitis, than those with low SES.^40^ In this study, SES was based on a combination of educational attainment and income. In the current study three measures to assess the association between HE and socioeconomic factors were chosen: educational attainment, income, and neighbourhood SES. We found an association between HE and higher educational attainment. However, no association was found for income and neighbourhood SES. The association between HE and higher educational attainment found in the current study may be explained by more awareness of HE, or skin diseases in general, in higher educated individuals. Furthermore, higher educated individuals may pay more attention to skin diseases and might, therefore, be more likely to report HE.^40^, ^41^ Overall, data on the association between socioeconomic factors and HE are conflicting and difficult to compare. This could be explained by differences in the categorization of income and education as well as the definition of SES. To further investigate the association between HE and socioeconomic factors, and to enable comparison between findings, a consensus should be reached on the definition of SES.

Some limitations to this study should be taken into account. First, the cross‐sectional data regarding HE is insufficient to determine causality between HE and socioeconomic and work‐related factors. Moreover, the data could have changed between baseline and the current add‐on study. This further complicates comparison with other studies because of variations in the study design. Furthermore, this may, for example, have led to the overestimation of HE prevalence in low income and low SES categories, underestimation in higher income categories, and distortion of HE prevalence among different occupational groups as a result of salary increases and/or changing of occupation over time. However, this was partly avoided by asking participants to report whether the onset of HE took place in certain high‐risk occupations.

The question regarding the 1‐year prevalence of HE used in the current study was validated by Meding et al., with a sensitivity and specificity of 65% and 93%, respectively.^42^ According to these results, the 1‐year prevalence of HE tends to be underestimated. On the other hand, as the non‐responder analysis showed that responders were more likely to be females and that females were more likely to report HE, the 1‐year prevalence of HE could be overestimated in the current study. As all data regarding the variables of interest were self‐reported, over‐ or underestimation of their true association with HE could be possible. Furthermore, the presence of selection bias could not be ruled out completely, especially because the invitation of the add‐on questionnaire was titled “Lifelines questionnaire: skin diseases”. Nevertheless, absolute differences between responders and non‐responders (except for age) were very small and were not considered as clinically relevant. A strength of this study is that it gives an overview of multiple socioeconomic and work‐related factors and their association with HE. In addition, both occupational and non‐occupational wet exposure were assessed. Other strengths are the large sample size and the large region covered in this study, namely the three Northern provinces of the Netherlands.

In conclusion, the most important findings of the current study were the association between HE and occupational as well as non‐occupational wet exposure. Positive associations for high‐risk occupations and occupations not considered as high‐risk (legal, social and religious, associate professionals) were found, as well as for higher levels of education. In daily practice, preventive strategies should focus on avoidance of all exposure to wet activities, regardless of origin. Special attention to occupations considered as high‐risk remains necessary, however, occupations not considered as high‐risk occupations should not be overlooked. Furthermore, to determine whether an individual is working in a high‐risk occupation, job tasks instead of job title should be considered. As previous results on the association between HE and socioeconomic factors differ between studies, future research should focus on a validated definition of SES for investigating the association with HE.

## AUTHOR CONTRIBUTIONS

**Marjolein Brands:** Conceptualization (equal); data curation (lead); formal analysis (lead); investigation (equal); methodology (equal); project administration (lead); resources (equal); validation (equal); visualization (lead); writing – original draft (lead); writing – review and editing (equal). **Laura Loman:** Conceptualization (supporting); data curation (supporting); investigation (equal); methodology (equal); resources (equal); supervision (supporting); validation (equal); writing – review and editing (equal). **Marie L.A. Schuttelaar:** Conceptualization (equal); investigation (equal); methodology (equal); resources (equal); supervision (lead); validation (equal); writing – review and editing (equal).

## CONFLICTS OF INTEREST

M.L.A. Schuttelaar received consultancy fees from Sanofi Genzyme and Regeneron Pharmaceuticals and is an advisory board member for Sanofi Genzyme, Regeneron Pharmaceuticals, Pfizer, LEO Pharma, Lilly. The other authors report no conflicts of interest.

## Supporting information


**Appendix S1.** Questions and response options including references

**Appendix S2.** Non‐responder analysis

**Appendix S3.**Data of missing values

## Data Availability

Data available on request from the authors.
