# Supplementary material for: Exposure and work‐related factors in subjects with hand eczema: Data from a cross‐sectional questionnaire within the Lifelines Cohort Study
Source: Contact Dermatitis. 2022 Mar 15;86(6):493–506. doi: 10.1111/cod.14066 (PMC9314613; doi:10.1111/cod.14066)
Supplement: Supplementary file 1 — Appendix S1. Questions and response options including references [file COD-86-493-s003.docx]

**Exposure and work-related factors in subjects with hand eczema: data from a cross-sectional questionnaire within the Lifelines Cohort study**

Marjolein J. Brands, Laura Loman, Marie L.A. Schuttelaar

**Online supplement S1.** Questions and response options including references

| **Topic** | **Question and response options** | **References** |
| --- | --- | --- |
| Age | What is your year of birth? | None. |
| Sex | What is your sex?   - Male (1) - Female (2) | None. |
| Lifetime prevalence of hand eczema | Hand eczema is a skin disease of the hands. Symptoms are redness, desquamation/dry skin and sometimes vesicles and/or fissures. Hand eczema can cause itch and/or pain.  Have you ever (now or in the past) had hand eczema?   - Yes (1) - No (2) | NOSQ-2002; question D1, modified.^1^ |
| 1-year prevalence of hand eczema | Have you had hand eczema in the past 12 months?   - Yes (1) - No (2) | NOSQ-2002; question D1, modified.^1^ |
| Atopic dermatitis | Have you ever been diagnosed with atopic dermatitis or atopic eczema by a doctor?   - Yes (1) - No (2) | Barbarot et al. 2018; question P1.^2^ |
| Contact allergy | Have you ever had an allergy test, by patch testing the skin on your back?   - Yes (1) - No (2) | BAMSE Q75, 76, 77, modified ^3^ |
|  | What were the results of the patch test?   - Positve (at least 1 positive reaction) (1) - Negative (no reactions to any of the tested allergens)(2) | BAMSE Q75, 76, 77, modified ^3^ |
| Occupational wet exposure | To define occupational wet exposure, three questions were asked. Occupational wet exposure was defined as minimally 2 or more from answer category 4, or 1 from answer category 5.  At work: On an average day how many hours do your hands come into direct contact with water, fluids and/or moist products? (E.g. during washing your hands)   - Never (1) - Less than 0.5 hours (2) - 0.5-1 hour (3) - 1-2 hours (4) - More than 2 hours (5)   At work: On an average day, how many hours do you wear gloves that are impermeable to fluids?   - Never (1) - Less than 0.5 hours (2) - 0.5-1 hour (3) - 1-2 hours (4) - More than 2 hours (5)   At work: On an average day, how often do you wash your hands?   - Never (1) - Less than 5 times (2) - 5 to 10 times (3) - 10 to 20 times (4) - More than 20 times (5) | Behroozy, Oosterhaven, TRGS ^4-6^ |
| Non-occupational wet exposure | To define non-occupational wet exposure, three questions were asked. Non-ccupational wet exposure was defined as minimally 2 or more from answer category 4, or 1 from answer category 5.  At home: On an average day how many hours do your hands come into direct contact with water, fluids and/or moist products? (E.g. during washing your hands)   - Never (1) - Less than 0.5 hours (2) - 0.5-1 hour (3) - 1-2 hours (4) - More than 2 hours (5)   At home: On an average day, how many hours do you wear gloves that are impermeable to fluids?   - Never (1) - Less than 0.5 hours (2) - 0.5-1 hour (3) - 1-2 hours (4) - More than 2 hours (5)   At home: On an average day, how often do you wash your hands?   - Never (1) - Less than 5 times (2) - 5 to 10 times (3) - 10 to 20 times (4) - More than 20 times (5) | Behroozy, Oosterhaven, TRGS ^4-6^ |
| High-risk occupations | Did you develop hand eczema during working in one of the following industries? (If you had an administrative position within the relevant industry, please do not check 'yes')   - None of these professions (1) - Agricultural workers / gardeners (2) - Bakers/pastry makers (3) - Beauty specialists/nail stylists (4) - Butchers/slaughterhouse workers (5) - Canning and fish processing industry workers (6) - Food industry (7) - Construction workers/carpenters (8) - Cooks/kitchen workers/vegetable processers (9) - Dental technicians (10) - Fitters (11) - Florists (12) - Hairdressers (13) - Healthcare workers (14) - Housekeepers/cleaners (15) - Metal surface processers (16) - Painters and varnishers (17) - Plasterers (18) - Tanners (19) - Tile setters and terazzo workers (20) - Print or paperindustry (21) - Textile, leather, fur or pelts industry (22) | NOSQ Q.D8, EDEN study. Oosterhaven et al., Diepgen 2003, OSD 2012, Guideline HE 2015, Skoet 2004, Halkier-Sorensen 1996, Schwensen 2013  ^1,6-11^ |
| ISCO-08 | If you have (had) a paid job, what is your last or current profession?  What is your current profession? |  |
|  | Please expound on your profession or position by describing your main work activities (1-3) |  |
| Educational attainment | What is the highest level of education you have finished?   - no education (did not finish primary school (1) - primary education (primary school, special needs primary school) (2) - lower or preparatory secondary vocational education (such as lts, leao, lhno, vmbo) (3) - junior general secondary education (such as mavo, (m)ulo, mbo-short, vmbo-t) (4) - secondary vocational education or work-based learning pathway (such as mbo-long, mts, meao, bol, bbl, inas) (5) - senior general secondary education, pre-university secondary education (such as havo, vwo, atheneum, gymnasium, hbs, mms) (6) - higher vocational education (suc as hbo, hts, heao, ‘kandidaats’ university education (e.g. bachelor)) (7) - university education (8) - other: (9) | ^12,13^ |
| Nett household income | What is the net income per month? (if you share a household, include the net income of your partner(s)   - less than € 750 (1) - € 750 - € 1000 (2) - € 1000 - € 1500 (3) - € 1500 - € 2000 (4) - € 2000 - € 2500 (5) - € 2500 - € 3000 (6) - € 3000 - € 3500 (7) - more than €3500 (8) - i do not know this (9) - i prefer not to answer that (10) |  |
| Employment status | Do you do paid work, even if that is only for one or a few hours a week?   - Yes (1) - No (2) |  |
|  | Which situation(s) applies/apply to you?   - i am unemployed/looking for a job (registered with the employment office - i am unfit for work - i am on national assistance benefit - i am full-time housewife/house husband - i go to school/i study - i am retired (age 65) - i took early retirement |  |
| Number of workhours | How many hours do you do paid work on average? (xx hours) |  |

1. Susitaival P, Flyvholm MA, Meding B, et al. Nordic Occupational Skin Questionnaire (NOSQ-2002): a new tool for surveying occupational skin diseases and exposure. *Contact Dermatitis.* 2003;49(2):70-76.

2. Barbarot S, Auziere S, Gadkari A, et al. Epidemiology of atopic dermatitis in adults: Results from an international survey. *Allergy.* 2018;73(6):1284-1293.

3. miljömedicin KIif. BAMSE Project – Questionnaire 16 years to the adolescent. .

4. Behroozy A, Keegel TG. Wet-work Exposure: A Main Risk Factor for Occupational Hand Dermatitis. *Saf Health Work.* 2014;5(4):175-180.

5. Arbeitsmedizin. BfAu. TRGS 401: Risks resulting from skin contact - identification, assessment, measures. 2008.

6. Oosterhaven JAF, Flach PA, Bültmann U, et al. Presenteeism in a Dutch hand eczema population-a cross-sectional survey. *Contact Dermatitis.* 2018;79(1):10-19.

7. Rossi M, Coenraads PJ, Diepgen T, et al. Design and feasibility of an international study assessing the prevalence of contact allergy to fragrances in the general population: the European Dermato-Epidemiology Network Fragrance Study. *Dermatology.* 2010;221(3):267-275.

8. Diepgen TL, Andersen KE, Chosidow O, et al. Guidelines for diagnosis, prevention and treatment of hand eczema. *J Dtsch Dermatol Ges.* 2015;13(1):e1-22.

9. Skoet R, Olsen J, Mathiesen B, Iversen L, et al. A survey of occupational hand eczema in Denmark. *Contact Dermatitis.* 2004;51(4):159-166.

10. Halkier-Sørensen L. Occupational skin diseases. *Contact Dermatitis.* 1996;35(1 Suppl):1-120.

11. Schwensen JF, Friis UF, Menné T, et al. One thousand cases of severe occupational contact dermatitis. *Contact Dermatitis.* 2013;68(5):259-268.

12. OECD EU, UNESCO Institute for Statistics ISCED 2011 Operational Manual: Guidelines for Classifying National Education Programmes and Related Qualifications. In:2015.

13. Nuffic. The education system of the Netherlands. 2011.
