# Supplementary material for: Exposure and work‐related factors in subjects with hand eczema: Data from a cross‐sectional questionnaire within the Lifelines Cohort Study
Source: Contact Dermatitis. 2022 Mar 15;86(6):493–506. doi: 10.1111/cod.14066 (PMC9314613; doi:10.1111/cod.14066)
Supplement: Supplementary file 2 — Appendix S2. Non‐responder analysis [file COD-86-493-s001.docx]

**Exposure and work-related factors in subjects with hand eczema: data from a cross-sectional questionnaire within the Lifelines Cohort study**

Marjolein J. Brands, Laura Loman, Marie L.A. Schuttelaar

**Online supplemental table S2.** Non-responder analysis

|  | **Total, n (%)**  (n=134037) | **Responders, n (%)** (n=57046) | **Non-responders, n (%)** (n=76991) | ***P*-value** |
| --- | --- | --- | --- | --- |
| **Age, mean +/- SD** | 52.8 +/- 12.5 | 55.8 +/- 12.2 | 50.5 +/-12.3 | **<.001** |
| **Age range n/ntotal (%)**  25-34 years  35-44 years  45-54 years  55-64 years  ≥65 years | 10902 (8.1)  23816 (17.8)  39089 (29.2)  37167 (27.7)  23063 (17.2) | 3080 (5.4)  7151 (12.5)  14704 (25.8)  18422 (32.3)  13689 (24.0) | 7822 (10.2)  16665 (21.6)  24385 (31.7)  18745 (24.3)  9374 (12.2) | **<.001**  **<.001**  **<.001**  **<.001**  **<.001** |
| **Sex**  Male  Female | 55585 (41.5)  78452 (58.5) | 22650 (39.7)  34396 (60.3) | 32935 (42.8)  44056 (57.2) | **<.001** |
| **Eczema** | 20645 (15.4) | 9051 (15.9) | 11594 (15.1) | **<.001** |
| **Psoriasis** | 3728 (2.8) | 1724 (3.0) | 2004 (2.6) | **<.001** |
| **Severe acne** | 3836 (2.9) | 1583 (2.8) | 2253 (2.9) | 0.10 |
| **SES**  Low  Middle  High | 39111 (36.7)  61451 (57.6)  6040 (5.7) | 15204 (34.8)  25869 (59.2)  2604 (6.0) | 23907 (38.0)  35582 (56.5)  3436 (5.5) | **<.001**  **<.001**  **<.001** |
| **Educational attainment**  Low  Middle  High | 36569 (27.8)  53549 (40.7)  41316 (31.4) | 14873 (26.5)  21969 (39.2)  19211 (34.3) | 21696 (28.8)  31580 (41.9)  22105 (29.3) | **<.001**  **<.001**  **<.001** |
| **Nett household income (euros per month)**  <750  750-1000  1000-1500  1500-2000  2000-2500  2500-3000  3000-3500  >3500 | 5561 (5.0)  3635 (3.3)  10120 (9.1)  15778 (14.2)  17563 (15.8)  19475 (17.5)  16979 (15.3)  21881 (19.7) | 1749 (3.6)  1299 (2.7)  3951 (8.2)  6844 (14.2)  7984 (16.6)  8593 (17.8)  7634 (15.9)  10088 (21.0) | 3812 (6.1)  2336 (3.7)  6169 (9.8)  8934 (14.2)  9579 (15.2)  10882 (17.3)  9345 (14.9)  11793 (18.8) | **<.001**  **<.001**  **<.001**  0.99  **<.001**  **0.020**  **<.001**  **<.001** |
| **Employment status**  Unemployed  Retired  Unfit for work  Employed | 12353 (9.5)  7218 (5.6)  2773 (2.1)  106112 (82.0) | 5672 (10.3)  4232 (7.7)  1186 (2.1)  44177 (79.9) | 7617 (10.3)  2986 (4.0)  1587 (2.1)  61935 (83.6) | 0.94  **<.001**  0.95  **<.001** |
| **Workhours (hours per week), mean +/- SD** | 30.7 **+/-**13.5 | 30.3 **+/-**13.1 | 31.0 **+/-** 13.7 | **<.001** |
| **Workhours (hours/week)**  1-8  9-16  17-24  25-32  33-40  >40 | 7237 (6.6)  11058 (10.1)  19343 (17.7)  20473 (18.8)  35251 (32.3)  15769 (14.4) | 3075 (6.7)  4473 (9.8)  8315 (18.2)  9103 (19.9)  14750 (32.3)  5986 (13.1) | 4162 (6.6)  6585 (10.4)  11028 (17.4)  11370 (17.9)  20501 (32.3)  9783 (15.4) | 0.28  **0.001**  **0.001**  **<.001**  0.87  **<.001** |
| **ISCO-08 major occupation groups**  Managers  Professionals  Technicians and associate professionals  Clerical support workers  Service and sales workers  Skilled agricultural, forestry and fishery workers  Craft and related trades workers  Plants and machine operators and assemblers  Elementary occupations  Armed forces occupations | 233 (0.2)  6382 (5.0)  30024 (23.4)  24024 (18.7)  15631 (12.2)  27267 (21.2)  3055 (2.4)  9773 (7.6)  4043 (3.1)  7926 (6.2) | 96 (0.2)  2844 (5.2)  14129 (25.7)  10739 (19.6)  7113 (13.0)  10969 (20.0)  1209 (2.2)  3373 (6.1)  1539 (2.8)  2888 (5.3) | 137 (0.2)  3538 (4.8)  15895 (21.6)  13285 (18.1)  8518 (11.6)  16298 (22.2)  1846 (2.5)  6400 (8.7)  2504 (3.4)  5038 (6.9) | 0.63  **0.003**  **<.001**  **<.001**  **<.001**  **<.001**  **<.001**  **<.001**  **<.001**  **<.001** |

Questions regarding eczema, psoriasis and severe acne were included in the baseline assessment.
Abbreviations: *n,* number; *SD*, standard deviation; *SES*, socioeconomic status; *ISCO-08,* International Standard Classification of Occupations 2008. For data on missing values see online supplement S3
