# Supplementary material for: Exposure and work‐related factors in subjects with hand eczema: Data from a cross‐sectional questionnaire within the Lifelines Cohort Study
Source: Contact Dermatitis. 2022 Mar 15;86(6):493–506. doi: 10.1111/cod.14066 (PMC9314613; doi:10.1111/cod.14066)
Supplement: Supplementary file 3 — Appendix S3.Data of missing values [file COD-86-493-s002.docx]

**Exposure and work-related factors in subjects with hand eczema: data from a cross-sectional questionnaire within the Lifelines Cohort study**

Marjolein J. Brands, Laura Loman, Marie L.A. Schuttelaar

**Online supplement S3.** Data of missing values

**Table 1.** Characteristics of the study population, stratified for sex.

Missing values: Age: males 0, females 0; Sex: males 0, females 0; SES: males 5354, females 8015; Educational attainment: males 412, females 581; Employment status: males 870, females 1208; Nett household income in euros per month: males 2941, females 5963; Workhours per week: males 4203, females 7141; ISCO-08 major occupation groups: males 882, females 1265; AD: male 333, females 676; Patch test: males 47, females 97; Occupational wet exposure: males 847, females 1664; Non-occupational wet exposure: males 251, females 417; Overall wet exposure: males 1013, females 1914; Prevalence HE lifetime: males 0, females 0; Prevalence HE 1-year: 9 males, 33 females; Daily hours of direct contact with water, fluids and/or moist products at work: males 705, females 1337; Daily hours of wearing gloves at work: males 659, females 1279; Frequency of hand washing at work: males 699, females 1352; Daily hours of direct contact with water, fluids and/or moist product at home: males 134, females 211; Daily hours of wearing gloves at home: males 135, females 197; Frequency of hand washing at home: males 126, females 189.

**Table 2.** Univariate and multivariate logistic regression analysis for the association between reporting hand eczema in the past year and wet exposure, socioeconomic, and occupational factors.

Missing values for 1-year prevalence: Sex: 0; Age: 0; Patch test: 6; AD: 196; Occupational wet exposure: 151; Non-occupational wet exposure: 64; Overall wet exposure: 198; SES: 1036; Educational attainment: 58; Nett household income in euros per month: 579; Employment status: 128; Workhours per week: 660; Daily hours of direct contact with water, fluids and/or moist products at home: 33; Daily hours of wearing gloves at home: 27; Frequency of hand washing at home: 34; Daily hours of direct contact with water, fluids and/or moist products at work: 119; Daily hours of wearing gloves at work: 107; Frequency of hand washing at work: 120.

Missing values for no HE (lifetime): Sex: 0; Age: 0; Patch test: 67; AD: 589; Occupational wet work exposure: 2112; Non-occupational wet work exposure: 498; Overall wet work exposure: 2413; SES: 11.410; Educational attainment: 860; Nett household income in euros per month: 7634; Employment status: 1802; Workhours per week: 9894. Daily hours of direct contact with water, fluids and/or moist products at home: 253; Daily hours of wearing gloves at home: 249; Frequency of hand washing at home: 224; Daily hours of direct contact with water, fluids and/or moist products at work: 1722; Daily hours of wearing gloves at work: 1651; Frequency of hand washing at work: 1734.

**Table 3.** The frequency of hand eczema in the past year, analysed in major occupational groups groups (classified on the 3-digit level of the ISCO-08 classification) with at least 75 cases of hand eczema in the past year.

Missing values: 2147 in total, 147 with HE in the last year, 1855 without HE ever.

**Table 4.** Proportion of participants reporting onset of hand eczema while working in high-risk occupations.

Missing values: 0
